# Prognostic Risk Model and Tumor Immune Environment Modulation of m5C-Related LncRNAs in Pancreatic Ductal Adenocarcinoma

**DOI:** 10.3389/fimmu.2021.800268

**Published:** 2021-12-08

**Authors:** Hao Yuan, Jinhui Liu, Li Zhao, Pengfei Wu, Guosheng Chen, Qun Chen, Peng Shen, Taoyue Yang, Shaoqing Fan, Bin Xiao, Kuirong Jiang

**Affiliations:** ^1^ Pancreas Center, Department of General Surgery, The First Affiliated Hospital of Nanjing Medical University, Nanjing, China; ^2^ Pancreas Institute, Nanjing Medical University, Nanjing, China; ^3^ Department of Gynecology, The First Affiliated Hospital of Nanjing Medical University, Nanjing, China

**Keywords:** lncRNA, m5C methylation, tumor immune microenvironment, PDAC, prognostic model

## Abstract

RNA methylation modification is a key process in epigenetics that regulates posttranscriptional gene expression. With advances in next-generation sequencing technology, 5-methylcytosine (m5C) modification has also been found in multiple RNAs. Long non-coding RNAs (lncRNAs) were proved to have a key role in cancer progression and closely related to the tumor immune microenvironment. Thus, based on the PDAC patients’ clinical information and genetic transcriptome data from the TCGA database, we performed a detailed bioinformatic analysis to establish a m5C-related lncRNA prognostic risk model for PDAC patients and discovered the relationship between the risk model and PDAC immune microenvironment. Pearson correlation coefficient analysis was applied to conduct a m5C regulatory gene and m5C-related lncRNA co-expression network. Expression of m5C-related lncRNAs screened by univariate regression analysis with prognostic value showed a significant difference between pancreatic cancer and normal tissues. The least absolute shrinkage and selection operator (LASSO) Cox regression method was applied to determine an 8-m5C-related lncRNA prognostic risk model. We used principal component analysis to indicate that the risk model could distinguish all the samples clearly. The clinical nomogram also accurately predicted 1-, 1.5-, 2-, and 3-year survival time among PDAC patients. Additionally, this risk model was validated in the entire group and sub-test groups using KM analysis and ROC analysis. Combined with the clinical characteristics, the risk score was found to be an independent factor for predicting the survival of PDAC patients. Furthermore, the association between the risk model and tumor immune microenvironment was evaluated *via* the ESTIMATE R package and CIBERSORT method. Consequently, the results indicated that immune cells were associated with m5C-related lncRNA risk model scores and had different distribution in the high- and low-risk groups. Based on all these analyses, the m5C-related lncRNA risk model could be a reliable prognostic tool and therapeutic target for PDAC patients.

## Introduction

Pancreatic ductal adenocarcinoma (PDAC) is a common malignant tumor of the digestive system, which is characterized by high degree of malignancy, easy recurrence, and metastasis (1). Recently, the incidence rate of PDAC has increased year by year. Due to the lack of early diagnosis methods, most patients were diagnosed at an advanced stage and lost the chance of radical resection (2). Lately, personalized molecular therapies targeting specific tumor-associated genes and the application of immune checkpoint inhibitors have effectually meliorated the prognostic effect of patients with advanced cancer (3). However, pancreatic cancer is characterized by high rates of drug resistance and metastasis due to its specific tumor microenvironment, current therapies have not effectively contributed to the prognosis of patients with PDAC, and researchers still need more specific biomarkers for developing more effective diagnostic and treatment strategies (4). Hence, it is essential to find new molecular biomarkers for early diagnosis of PDAC.

Epigenetics study aims to research gene differential expression without changing nucleotide sequence (5), and epigenetic modification has been proven to play an important role in the progress of tumor biology (6), such as DNA and RNA methylation, genomic imprinting, gene silencing, and non-coding RNA modification. In recent years, N6-methyladenine (m6A) and 5-methylcytosine (m5C) RNA methylation have become a vital direction in detecting the regulation of RNA epigenetic modification upon the growth of various malignant tumors (7, 8). With the advance of high-throughput sequencing technology, the distribution and biological functions of RNA m5C modification at mRNA and non-coding RNA have been gradually discovered (9). RNA m5C methylation is catalyzed by a methyltransferase complex, which consists of three methylation-related enzymes, including methyltransferase (“Writer”), demethylase (“Erase”), and m5C binding protein (“Reader”). Similar to functions of m6A methylation, m5C methylation exerted biological effects mainly by regulating RNA stability, affecting transcription efficiency, and mediating RNA localization (10). In addition, abnormal expression of m5C methylation is associated with the occurrence and development of several malignant tumors (11, 12). Recent reports have also demonstrated that m5C-related gene expression is associated with prognosis in patients with lung and pancreatic cancers, which further indicated that m5C methylation may have an essential role in malignant tumor growth (13, 14).

Long non-coding RNAs are transcribed by corresponding genes and have a similar structure to mRNA including polyA tail and promoter structure (15). Due to different splicing methods, multiple lncRNAs were formed during the differentiation process. Several research unveiled that lncRNAs could modulate downstream genes in epigenetic, transcriptional, and post-transcriptional levels including gene silencing, histone processing, transcriptional regulation, transcriptional interference, and nuclear transport, which were closely related to the development of various human diseases (16). In malignant tumors, methylation-related genes could affect tumor progression by regulating the methylation level of lncRNA. For instance, LINC00942-specific sequence recruited methylated transferase METTL14 and stabilized the downstream targets of LNC942, which could promote tumorigenesis and development in breast cancer (17). Several reports have demonstrated that m5C modification existed widely in non-coding RNAs; nevertheless, the reports about m5C methylation regulation in lncRNAs were still few. Thus, it is essential to further research the relationship between m5C methylation and lncRNAs, especially in malignant tumor.

Tumor microenvironment (TME) is a mixture of fluid, immune cells, and blood vessels surrounding the tumor (18). The interaction between tumor cells and TME could determine the progression and fate of tumor. Therefore, understanding the composition and function of TME is crucial to research cancer progression (19). Studies have indicated that multiple genetic mutations could increase the incidence of cancer. However, the impact of TME on cancer development is still unclear. Accumulating evidence revealed that lncRNAs were important regulators in the immune system, which could control the distribution and activation of immune cells and mediate the tumor cells’ immune evasion. For instance, LncRNA-MALAT1 could promote thyroid cancer progression *via* modulating tumor-associated macrophages secreting FGF2 protein (20). In colorectal cancer, lncRNA SATB2-AS1 could regulate tumor tissue immune cell density *via* combining with downstream proteins WDR5 and GADD45A and regulating TH1-type chemokines (21). Furthermore, the relationship between lncRNAs and tumor immune microenvironment has also been reported in pancreatic cancer (22). However, the relationship between m5C-related lncRNAs and tumor-infiltrating lymph cell distribution has never been reported in PDAC.

In our study, we summarized 177 PDAC patients’ clinical information together with transcriptome expression data, and constructed an mRNA–lncRNA co-expression network between m5C-related mRNAs and lncRNAs. A total of 17 prognostic lncRNAs were screened *via* univariate Cox analysis. Consequently, we generated a PDAC prognostic risk model consisting of 8 m5C-related lncRNAs *via* LASSO regression analysis. We further verified the prognostic risk model in subgroups *via* Kaplan–Meier (KM) analysis and receiver operating characteristic curve (ROC) analysis. Additionally, the association between the risk model and tumor immune environment was measured *via* the ESTIMATE R package and CIBERSORT tool. Finally, 6 types of immune cells were identified in high- or low-risk groups. Moreover, m5C-related lncRNAs had statistically significant association with immune related genes and 5 types of tumor-infiltrating lymph cells were negatively correlated with the risk scores. In summary, our research indicated that the m5C-related lncRNA risk model might offer a promising prognostic tool and play an essential role in regulating PDAC immune cell distribution.

## Materials and Methods

### Data Collection and Processing

We downloaded a total of 182 patient data from the TCGA data website including clinical and transcriptome expression raw data (https://portal.gdc.cancer.gov/repository). Compared with the Ensemble Genes ID, 14,086 lncRNAs were identified in the TCGA dataset. Online bioinformatic tool GEPIA (http://gepia.cancer-pku.cn/index.html) was used to detect the expression of m5C-related genes in pancreatic normal and tumor tissues (|Log2FC| Cutoff = 1, *p*-value cutoff = 0.01; Match TCGA normal and GTEx data).

### LASSO Analysis

The LASSO regression method was introduced to construct a m5C regulatory gene-related lncRNA predicted risk model *via* R package. It is a widely used high-dimensional index regression method that screened the m5C-related lnRNAs with prognostic value and constructed a predicted risk model by applying a penalty proportional to the contraction of the regression coefficient. We established a m5C-related lncRNA risk model consisting of 8 lncRNAs depending on this method.

### GSEA

We divided all samples into two groups according to the median risk score of the m5C-related lncRNAs. GSEA software (GSEA_4.1.0) was performed to analyze the data. Enrichments of gene sets with a normalized *p*-value less than 0.05 were considered to be significant.

### Cell Culture

HPNE cell lines (human pancreatic ductal epithelial cell line) and all the three human pancreatic cancer cell lines (Mia-PaCa-2, Panc-1, and CFPAC-1) were purchased from the Cell Bank of Type Culture Collection of the Chinese Academy of Sciences in Shanghai, China. The cells were cultured in RPMI 1640 medium (Gibco, United States) or Dulbecco’s modified Eagle’s medium (Gibco, United States) with 10% fetal bovine serum (Wisent, Montreal, QC, Canada), 100 U/ml penicillin, and 100 μg/ml streptomycin in the incubator at 37°C and 5% CO_2_ concentration.

### RNA Isolation and Quantitative Real-Time PCR

Total RNA was isolated from pancreatic cancer cells and HPNE cells by Trizol reagent (Life Technologies, Carlsbad, CA, USA) based on the manufacturer’s instruction. After quality validation, iScript cDNA Synthesis Kit (Bio-Rad, Hercules, CA, USA) was used to perform reverse transcription. Total cDNA was further used for qRT-PCR with the TaqMan Gene Expression Assay (Thermo Fisher Scientific, Rockford, IL, USA). The expression of GAPDH was used as an internal control to calibrate the original mRNA expression level. The m5C-related lncRNA expression was calculated *via* the 2^-ΔΔCT^ method. All primer sequences used in this research are listed in **Supplementary Table 1**.

### Identification Tumor-Infiltrating Immune Cells

CIBERSORT is a typical tool to deconvolute the expression matrix of immune cells based on linear support vector regression, which quantified the infiltrating immune cells proportion *via* detecting marker gene expression. In our study, we combined the expression of marker genes from 22 types of immune cells and all the PDAC patients’ transcriptome data to acquire the tumor-infiltrating lymph cell distribution scores *via* CIBERSORT. ESTIMATE R package could use gene expression profile to predict stromal cell and immune cell scores, and then calculate the amount of these two types of cells, which was performed to analyze the pancreatic cancer purity in this research.

### Statistical Analysis

The data flow chart is shown in **Supplementary Table 2**. The m5C-related lncRNAs with correlation index > 0.3 and *p* < 0.05 were regarded as statistically significant. Univariate and multivariate Cox regression analyses were performed to identify the m5C-related lncRNA risk score as an independent prognostic factor for PDAC. The Kaplan–Meier method was performed to compare OS time of PDAC patients. ROC analysis was expended to measure the prognostic competence between the prognostic risk scores and other clinical parameters. The qRT-PCR experiments were analyzed by PRISM 7. The relationship between the m5C-related lncRNA risk score and tumor-infiltrating lymph cell distribution was analyzed by CIBERSORT and ESTIMATE R package.

## Results

### Identification of m5C Regulator-Related LncRNAs in PDAC Patients

**Figure 1:**
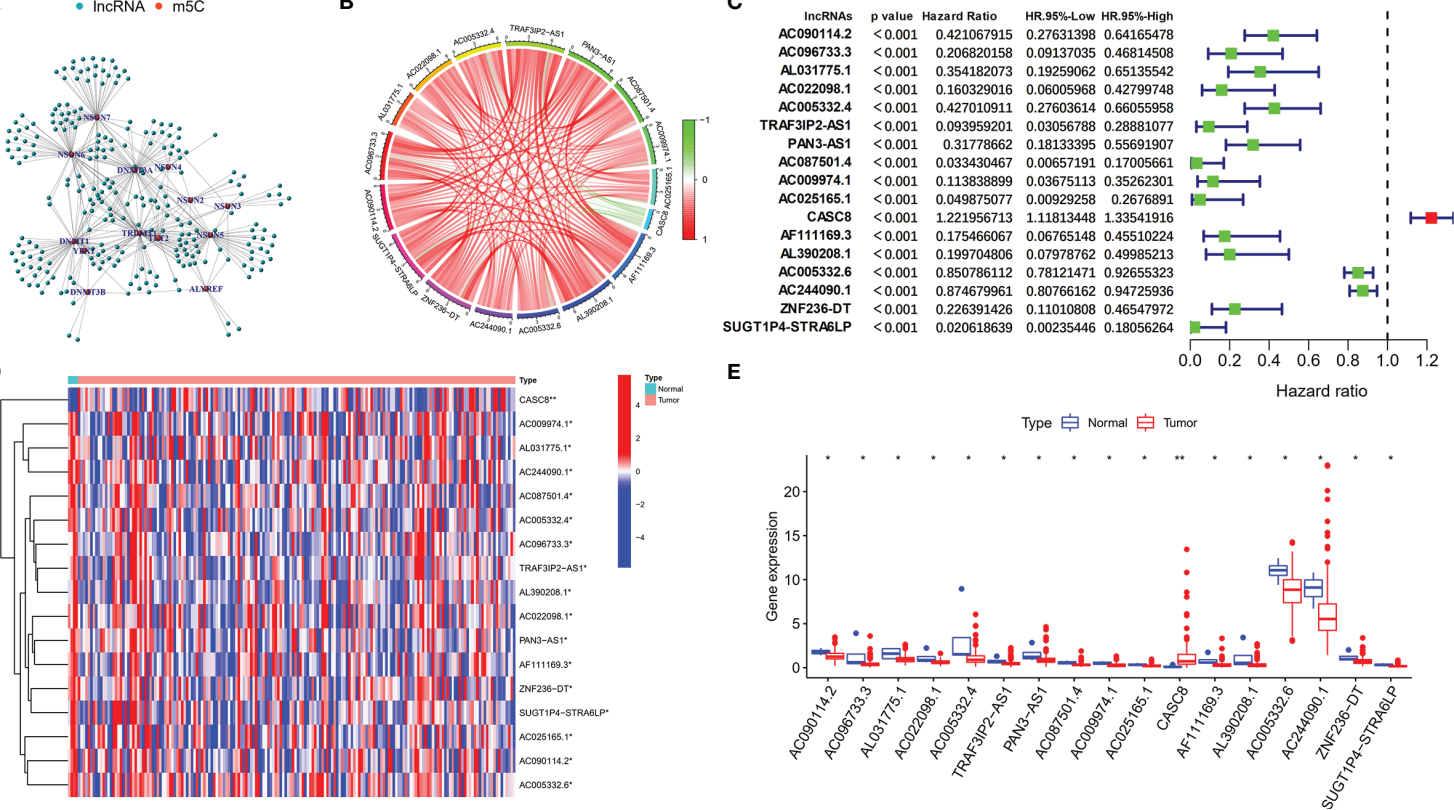
Identification of m5C regulator-related lncRNAs with prognostic value in PDAC patients. **(A)** Co-expression relationship between m5C-related lncRNAs and m5C regulators. **(B)** Circos figure exhibited the expression correlation between the m5C-related lncRNAs with prognostic value (red line represents positive relationship and the green line represents negative relationship). **(C)** Forest plot showed the prognostic risk value of the 17 screened m5C-related lncRNAs *via* univariate Cox regression analysis. **(D, E)** Heatmap and scatter diagram indicated the different expression of m5C-related lncRNAs in normal and tumor tissues. *p < 0.05, **p < 0.01.

Initially, we screened the m5C methylation modification-related genes based on the published articles. A total of 13 m5C regulators were selected, namely, YBX1, ALYREF, DNMT1, NSUN4, TRDMT1, TET2, NSUN7, NSUN6, NSUN5, NSUN3, NSUN2, DNMT3a, and DNMT3b. We summarized the m5C regulator genes and then compared the expression of m5C regulator genes in 179 normal pancreas tissues and 171 pancreatic cancer tissues using online data base GEPIA (http://gepia.cancer-pku.cn/). We found that among the m5C regulators, the expression of YBX1, ALYREF, DNMT1, and NSUN4 was obviously higher in PDAC patient’s tissues (*p* < 0.05), whereas no notable variances were detected in TRDMT1, TET2, NSUN7, NSUN6, NSUN5, NSUN3, NSUN2, DNMT3a, and DNMT3b (**Supplementary Figure 1**). Moreover, the m5C-related lncRNAs were recognized by correlation analysis depending on the expression of m5C regulators and lncRNAs in a total of 177 PDAC samples. Use correlation coefficient > 0.3 and *p*-value > 0.05 as filter criterion; finally, 242 m5C-related lncRNAs were screened. We also constructed a network between m5C-related genes and their co-expressing lncRNAs to show m5C-related lncRNA co-expressing relationship (**Figure 1A**). Based on the screened m5C-related lncRNAs, we use univariate Cox analysis (*p* < 0.001) to further filter the prognostic m5C-related lncRNA in PDAC patients combined with the patient survival data. The results indicated that a total of 17 PDAC prognostic lncRNAs were screened, and most of the m5C-related lncRNAs were protective factors (hazard ratio, HR < 1), only CASC8 was a risk factor (HR > 1) in PDAC. In addition, the Circos picture showed that the protective m5C-related lncRNAs had positive expression correlation and risk factor CASC8 was negatively correlated with others (**Figures 1B, C**). Furthermore, we tried to clarify the expression of the screened prognostic m5C-related lncRNAs in PDAC patients depending on the TCGA database. The heatmap revealed that all the lncRNAs had statistical differences between the normal and tumor pancreatic tissues, which indicated that the m5C-related lncRNAs might play a key role in PDAC progression (**Figures 1D, E**).

### Establishing an 8 m5C-Related LncRNA Risk Model for PDAC Patients

**Figure 2:**
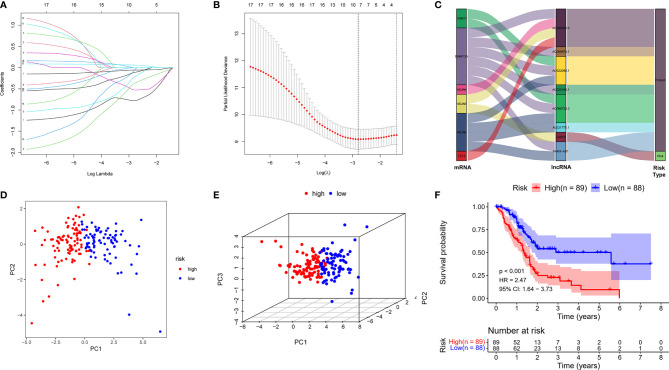
Constructed a m5C-related lncRNA risk model in the PDAC cohort. **(A)** LASSO regression of the 17 m5C-related lncRNAs. **(B)** Cross-validation for optimizing the parameter in LASSO regression. **(C)** The Sankey diagram displayed the relationship between the 6 m5C regulators mRNA expression and the 8 m5C-related lncRNAs. **(D, E)** PCA and three-dimensional PCA analysis derived from the m5C-related lncRNAs indicated that the patients were divided into two significantly high- or low-risk distribution patterns. **(F)** KM curve shows that patients in the m5C-related lncRNA low-risk group survived dramatically longer than those in the high-risk group.

Depending on the primary screen of the m5C-related lncRNAs, the LASSO regression technique was further performed randomly to construct a prognostic risk model for PDAC, which showed that eight m5C-related lncRNAs were appropriate for building the prognostic risk model (**Figures 2A, B** and **Table 1**). The risk score was calculated as follows: risk score = (-0.780839063578865 * AC022098.1. expression) + (-0.220638925265728 * AL031775.1 expression) + (-0.0579614996945241 * AC005332.6 expression) + (-0.367271578600146 * AC096733.3 expression) + (-0.0490022123517058 * AC025165.1 expression) + (0.0600252490282948 * CASC8 expression) + (-0.528438814531151 * AC009974.1 expression) + (-0.113641932851614 * PAN3-AS1 expression). The Sankey diagram indicated the relationship between the 6 m5C regulators mRNAs and 8 screened lncRNAs, of which CASC8 belonged to risk factors and the others were protective factors (**Figure 2C**). Furthermore, all the PDAC patients were divided into low- or high-risk groups based on the median risk scores calculated by the above formula. The principal component analysis (PCA) and three-dimensional PCA showed that patients with different risks were well separated into two clusters (**Figures 2D, E**). Patients in the high-risk group had significantly shorter survival time than the patients in the low-risk group. A distinguished difference in OS time was detected between the low- and high-risk groups *via* Kaplan–Meier survival analysis (HR = 2.47, 95% CI: 1.64–3.73, *p* < 0.001, **Figure 2F**).

**Table 1:** The 8 m5C-related lncRNA risk model parameters.

LncRNAs	AC022098.1	AL031775.1	AC005332.6	AC096733.3	AC025165.1	CASC8	AC009974.1	PAN3-AS1
Correlation coefficient	-0.780839064	-0.220638925	-0.0579615	-0.367271579	-0.049002212	-0.060025249	-0.528438815	-0.113641933

### Relationships Between m5C-Related LncRNAs and Clinical Pathological Parameters

**Figure 3:**
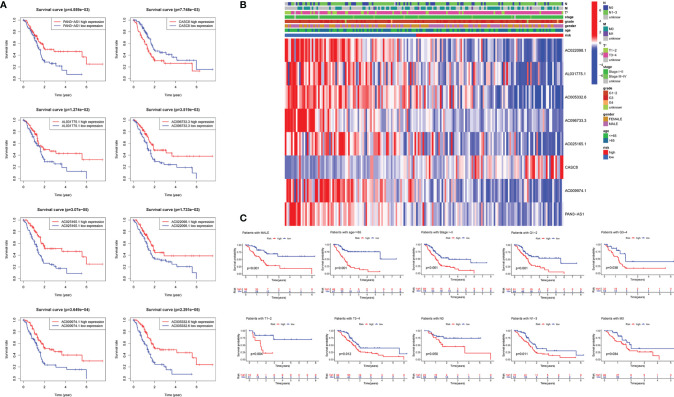
Relationships between m5C-related lncRNAs and clinical pathological parameters. **(A)** Eight survival curves based on the m5C-related lncRNAs expression. **(B)** Heatmap displayed the clinical characteristics and differences in the high- and low-risk group calculated by m5C-related lncRNA risk scores. **(C)** Survival analysis in subgroups including gender, age and tumor stages.

We further detected the impact of the selected eight lncRNAs on PDAC patients’ overall survival time, respectively. The OS curve of the m5C-related lncRNAs showed that patients with risk lncRNA high expression (CASC8) had shorter survival time, whereas those with protective lncRNA high expression (AC022098.1, AL031775.1, AC005332.6, AC096733.3, AC025165.1, AC009974.1, and PAN3-AS1) had longer survival time (**Figure 3A**). According to the heatmap, the pancreatic tumor size was significantly different between the high- and low-risk groups (*p* < 0.05). Nevertheless, the other clinical factors including patient age, gender, tumor stage, and grade had no statistical differences (**Figure 3B**). Moreover, we further subdivide these clinical indicators severally and analyzed the risk scores in the subgroups. The KM survival curve showed that the patients with high-risk scores had a shorter OS in male patients’ group, younger patients (age less than 65 years), Stage I–II groups, Grade I–II or Grade III–IV groups, T I–II or T III–IV groups, N0 or N I–III groups, and patients without any metastasis group (**Figure 3C** and **Supplementary Figure 2**).

### The m5C-Related LncRNA Risk Score Was an Independent Prognostic Factor for PDAC

**Figure 4:**
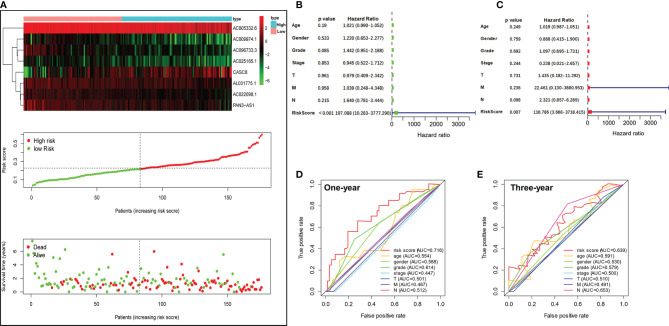
Valuation of the m5C-related lncRNA risk model as an independent prognostic factor for PDAC. **(A)** Heatmap showed the differential expression of the 8 m5C-related lncRNAs in the high- or low-risk group. Scatter plot displayed risk score distribution of high-risk and low-risk PDAC patients based on the m5C-related lncRNA risk model and the relationship between survival time and PDAC patients risk score. **(B)** Univariate Cox regression analysis revealed the association between patients’ survival and clinicopathological parameters along with m5C-related lncRNA risk score. **(C)** Multivariate Cox regression analysis uncovered that only the risk score (*p* = 0.009) was an independent prognostic factor for PDAC patients. **(D, E)** The 1-year **(D)** and 3-year **(E)** ROC analysis revealed the AUCs of m5C-related lncRNA risk score and other clinical characteristics.

We ordered the patients based on the risk scores. Heatmap revealed that the expression of 8 m5C-related lncRNAs were considerably different in the PDAC patients with different risk scores. Furthermore, the scatter dot plot also unveiled that the mortality of the PDAC patients increased with the risk scores, and patients with lower risk scores exhibited longer survival time (**Figure 4A**). Next, we performed univariate and multivariable Cox regression analyses to validate if the risk score calculated by the m5C-related lncRNA risk model could work as an independent prognostic factor for PDAC patients. As shown in **Figure 4B**, univariate Cox regression analysis demonstrated that only the m5C-related lncRNA risk scores were obviously positively related with OS (HR: 197.088, 95% CI: 10.283–3777.290, *p* < 0.001). Multivariate analysis also revealed that m5C-related lncRNAs prognostic risk score (HR: 116.786, 95% CI: 3.668–3718.415, *p* = 0.007) had a significant relationship with PDAC patients’ OS and could act as an independent prognostic factor (**Figure 4C**). Our results suggested that the m5C-related lncRNA risk model was an independent prognostic factor for PDAC and took advantage of the traditional clinicopathological indicators including patient age, gender, and tumor stage. Moreover, the 1-year ROC curve proved that the AUC value for the m5C-related lncRNA risk model was 0.716, which was better than the traditional clinical factors such as age (AUC = 0.554), gender (AUC = 0.588), grade (AUC = 0.614), AJCC stage (AUC = 0.447), T stage (AUC = 0.447), N stage (AUC = 0.512), and M stage (AUC = 0.467) (**Figure 4D**). In addition, the 3-year ROC curve analysis also suggested that the risk score AUC value (AUC = 0.639) was a superior prognostic factor for PDAC patients (**Figure 4E**).

### Exanimating the Prediction Value of the m5C-Related LncRNA Risk Model

**Figure 5:**
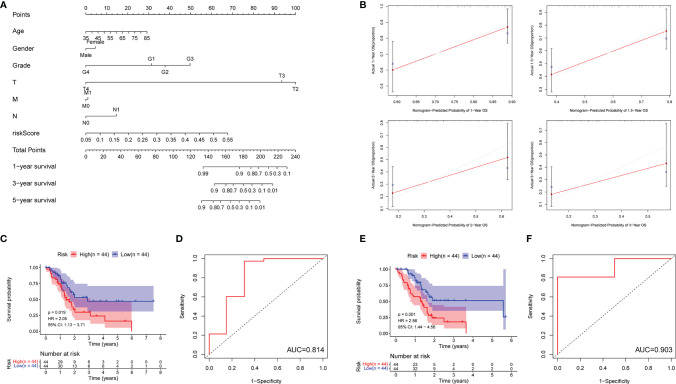
Detecting the prediction value of the m5C-related lncRNA risk model. **(A)** The prediction of 1-, 3-, and 5-year survival for PDAC patients based on the prognostic nomogram derived from the m5C-related lncRNA risk score and other clinicopathologic feature. **(B)** Calibration curves illustrated the consistency between predicted and observed 1-, 1.5-, 2-, and 3-year survival rates in PDAC patients depending on the prognostic nomogram. **(C–F)** Overall survival and ROC analysis in subgroups (**C**, **D**: Group **A**; **E**, **F**: Group **B**).

In order to predict the PDAC patient overall survival accurately, a nomogram was constructed to reveal the 1-, 3-, and 5-year survival rates based on the m5C-related lncRNAs expression risk scores together with clinicopathologic features (including age, gender, grade, T stage, M stage, and N stage) (**Figure 5A**). In addition, we used a calibration curve to compare the consistency of the actual and the predicted 1-, 1.5-, 2-, and 3-year patient survival. We found that the actual and the predicted line were almost in accordance within 3 years (**Figure 5B**). The above results proved that the nomogram we generated *via* m5C-related lncRNA prognostic risk scores was dependable. In addition, we randomly divided all the PDAC patients from TCGA into two subgroups (group A and B) in a 1:1 ratio and performed an internal validation for the m5C-related lncRNA risk model. The KM survival curve and 5-year ROC curve were examined in each subgroup. Our results showed that the patients with higher m5C-related lncRNA risk scores had shorter OS in group A (HR: 2.05, 95% CI: 1.13–3.71, *p* = 0.019) and the AUC value of the 5-year ROC curve was 0.814 (**Figures 5C, D**). The patients in group B had a similar OS trend (HR: 2.56, 95% CI: 1.44–4.56, *p* = 0.001) and the AUC value was 0.903 (**Figures 5E, F**). Moreover, the scatter dot plot also revealed an obvious difference expression of the screened lncRNAs in high- or low-risk groups, and the patient survival time was positively related with the risk scores in all the subgroups (**Supplementary Figure 3**). The above results demonstrated that the m5C-related lncRNA risk model was a reliable predictive factor for the PDAC patient.

### Detecting LnRNA Expression *In Vitro* and Functional Enrichment Analysis

**Figure 6:**
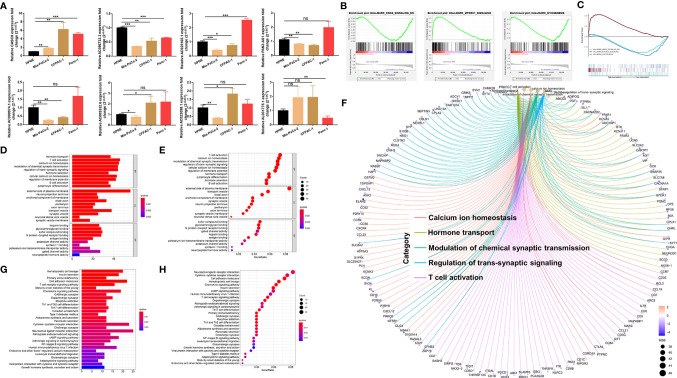
Validating the expression level of the m5C related lncRNAs *in vitro* and functional enrichment analysis. **(A)** qRT-PCR experiments were performed to detect the expression of the 8 m5C related lncRNAs in HPNE cells and three pancreatic cancer cells respectively. **(B, C)** GSEA results showed significant enrichment signaling pathways in the high-and low-risk groups. **(D–F)** GO analysis was performed to detect biological processes that involved in the high-or low-risk groups. **(G, H)** KEGG pathway analysis results indicated the enriched signaling pathways associated with the m5C related lncRNAs risk scores. ns, not significant, *P < 0.05, **P < 0.01, ***P < 0.001.

The m5C-related lncRNA expression profiles from the TCGA database indicated that AC022098.1, AL031775.1, AC005332.6, AC096733.3, AC025165.1, AC009974.1, and PAN3-AS1 were downregulated, and the CASC8 was overexpressed in PDAC. In order to examine the lncRNAs’ expression level *in vitro*, three pancreatic cancer cell lines and a normal pancreatic duct cell line were used to perform the qRT-PCR experiments. The *in vitro* results were not completely consistent with the data from TCGA (**Figure 6A**). We found that CASC8 was upregulated in pancreatic cancer cells (Mia-PaCa-2, CFPAC-1, and Panc-1 cells) and AC096733.3 was in low expression compared with HPNE cells, which were with the same expression profile with TCGA data. AC096733.3, AC025165.1, PAN3-AS1, and AC009974.1 were downregulated in at least two cancer cell lines and may play as tumor suppressor genes in PDAC. The above results were partly consistent with the patients’ OS analysis data from TCGA. However, AC022098.1 and AC005332.6 were suppressed in Mia-PaCa-2 and overexpressed in CFPAC-1, and their expression was inconsistent in different cells. In addition, AL031775.1 was only downregulated in Panc-1 cells. Hence, the underlying mechanisms that m5C-related lncRNAs regulating PDAC patient OS time still needs to be further explored. To seek the signal pathways that m5C-related lncRNAs might involve in the low- or high-risk groups, GSEA was performed. We found that the MTORC1 signaling pathway was activated in the high-risk group; nevertheless, MYOGENESIS and KRAS signaling pathways were activated in the low-risk group (**Figures 6B, C**). Furthermore, we summarized differential genes in the low- or high-risk groups to conduct GO and KEGG enrichment analysis. The top five GO enrichment biological processes were T-cell activation, Calcium ion homeostasis, Hormone transport, Modulation of chemical synaptic transmission, and Regulation of trans-synaptic signaling pathway (**Figures 6D**–**F**). Additionally, KEGG analysis revealed that several immune-related pathways were enriched such as Primary immunodeficiency, T-cell receptor signaling pathway, Leukocyte transendothelial migration, and Th1/Th2 cell differentiation (**Figures 6G, H**).

### The Relationship Between m5C-Related LncRNAs and Tumor-Infiltrating Lymph Cells

**Figure 7:**
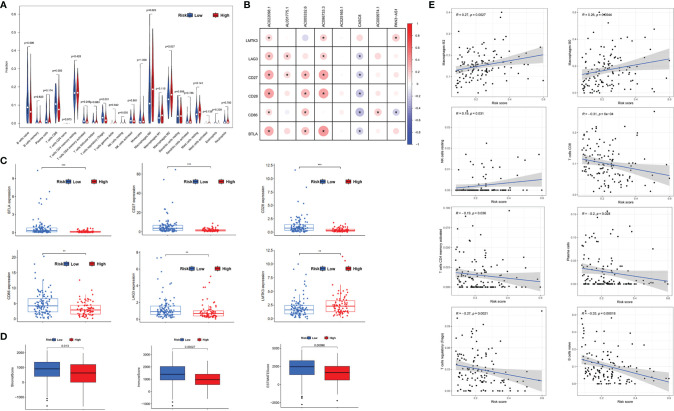
The relationship between m5C-related lncRNAs and tumor-infiltrating lymph cells. **(A)** Violet plot showed the 22 tumor-infiltrating lymph cells distribution in PDAC patients with different m5C-related lncRNA risk scores. **(B)** The connection between the immune associated genes and the selected m5C-related lncRNAs. **(C)** Scatter diagram revealed the expression of immune-related genes in low- or high-risk groups, respectively. **(D)** Box plots indicated the proportion of stromal and immune cells in PDAC tissues *via* ESTIMATE R package method. **(E)** Correlation between the lymph cells and m5C-related lncRNA risk scores. *p < 0.05, **p < 0.01, ***p < 0.001.

We analyzed the differences of tumor microenvironment in the high- or low-risk groups. By using CIBERSORT tool with values of *p* < 0.05, a total of 22 tumor-infiltrating immune cells were screened (**Figure 7A**). The results demonstrated that naïve B cells, CD8^+^T cells, regulatory T (Treg) cells, and resting NK cells exhibited a higher expression in the low-risk group (*p* < 0.05), whereas the M0 and M2 phenotype macrophages had a higher expression in the high-risk group. M2 phenotype macrophage has been proven as a co-carcinogenic factor in pancreatic cancer. Hence, m5C-related lncRNAs may promote M2 phenotype macrophage polarization or infiltration in PDAC. Additionally, we further detected the relationship between m5C-related lncRNAs and immune-related genes (LMTK3, LAG3, CD27, CD28, CD86, and BTLA), which indicated that most of the lncRNAs had statistical correlation with immune-related genes except AC025165.1 (*p* < 0.05) (**Figure 7B**). Gene expression analysis also showed that expression of the above immune-related genes are different in low- or high-risk groups (**Figure 7C**). Moreover, tumor microenvironment scores were performed to analyze the stromal and immune cell proportion in the tumor environment *via* ESTIMATE R package. We obviously found that the PDAC tissues with low m5C-related lncRNA risk scores had higher immune scores, stromal scores, and overall ESTIMATE scores, which indicated that the PDAC in the low-risk group had lower pancreatic tumor proportion (**Figure 7D**). Lastly, we also investigated the association between the m5C-related lncRNA risk score and tumor lymph cells. Spearman’s correlation analysis revealed that the risk score was positively related with 3 tumor-infiltrating lymph cells (NK cells, M0, and M2 phenotype macrophages), but was negatively correlated with regulatory T cells, CD8^+^T cells, activated memory CD4^+^T cells, naïve B cells, and plasma cells (*p* < 0.05) (**Figure 7E**). These outcomes revealed that the m5C-related risk scores could discriminate the different features of tumor-infiltrating lymph cells in PDAC.

## Discussion

Pancreatic cancer is an extremely malignant cancer, and its incidence is close to mortality rate. In recent years, although comprehensive treatments based on surgery have made some progress in the treatment of PDAC patients, the diagnosis and prognosis of PDAC are still unsatisfactory, and the survival rate is still less than 10%. Early diagnosis is the key to the treatment of pancreatic cancer (23). However, the etiology of pancreatic cancer is still unclear and there is a lack of effective biomarkers for early diagnosis. Therefore, it is urgent to explore the genetic or epigenetic factors of the occurrence and development of pancreatic cancer to identify new therapeutic targets or biomarkers.

RNA methylation is one of the most important epigenetic modification in RNA post-transcriptional regulation and has been a research frontier and hotspot recently. RNA methylation includes several types such as m6A, m1A, m5C, m7G, and Nm, among which RNA m5C methylation refers to the modification of the fifth cytosine of RNA by methylation (24–26). At present, relevant studies have shown that RNA m5C methylation widely existed in cells and played an important role in various physiological and pathological processes (27–29). In this study, we downloaded 177 PDAC patients’ gene expression profiles from the TCGA database and constructed an 8-m5C-related lncRNA risk model. As far as we know, this is the first prognostic analysis of the m5C regulator-related lncRNAs in PDAC.

Long non-coding RNAs are more than 200 nucleotides, which were initially considered as “junk sequence” without specific biological functions. However, recent reports have proved that lncRNAs were widely expressed in human cells and closely related to the occurrence and development of tumors, which suggested that lncRNAs may be potential prognostic markers and therapeutic targets for malignant tumors (30). In addition, lncRNAs could regulate tumor growth in several ways including *in situ* regulation and molecular sink, combining with protein and molecular scaffold. Among them, the most essential way was regulating related gene expression (31, 32). Hence, we focused on the lncRNAs that had co-expression relationship with m5C regulators in PDAC and used bioinformatic and statistical methods to generate a prognostic risk model for PDAC.

In our research, we compared the expression of 13 m5C regulator genes in pancreatic cancer and normal tissues *via* the GEPIA online tool and identified a total of 242 m5C-related lncRNAs depending on the co-expression relationship. Furthermore, we analyzed the PDAC patients’ clinical information together with gene transcriptome data downloaded from TCGA. Finally, 17 prognostic m5C-related lncRNAs were detected and all the prognostic m5C-related lncRNA expression was significantly different between pancreas normal and tumor tissues. In addition, LASSO regression was performed, and we constructed an 8-m5C-related lncRNA risk model and separated all the 177 PDAC patients into high- and low-risk groups based on the risk scores. The PCA and patients’ overall survival analysis confirmed that the 8-m5C-related lncRNA risk model could predict PDAC patient prognosis effectively. Typically, clinicians would make a rough judgment on PDAC patients’ long-term survival based on clinical indicators such as general patient profile, pancreatic tumor size, invasion of peripheral blood vessels and nerves, presence of metastasis, and tumor pathological type. However, with advances in cancer gene research technology, scientists began to pay more attention to the changes in gene expression levels during tumor progression (33, 34). In our work, univariate and multivariate Cox regression analysis indicated that the risk scores depending on the m5C-related lncRNAs expression were the independent risk factor for PDAC and better than traditional indexes including age, tumor grade, and stage on predicting patient survival rate. The currently constructed PDAC prognostic risk model mainly focused on a single molecular biomarker. However, only one biomarker could not work as a precise tumor biomarker in clinical diagnosis such as the typical PDAC biomarker CA19-9 that was not aberrant in every PDAC patient. It has been reported that lncRNAs could be released in serum, saliva, and urine (35). Hence, it has the potential to be a stable biomarker in human blood or other body fluid. A lot of studies have demonstrated that lncRNAs could work as a new type of biomarker in several malignant tumors including hepatocellular carcinoma, breast cancer, lung cancer, and pancreatic cancer (36). Nevertheless, our research was the first study to report an 8-prognostic m5C-related lncRNA risk model and validated that the prognostic risk model could predict PDAC patients’ OS accurately. In our research, 8 m5C-related lncRNAs were carefully chosen *via* a bioinformatic method, 7 of which have never been previously studied in PDAC (AC022098.1, AL031775.1, AC005332.6, AC096733.3, AC025165.1, AC009974.1, and PAN3-AS1). Only the CASC8 has been reported in the multiple malignant tumor such as colorectal cancer, lung cancer, and hepatocellular carcinoma (37–39). We checked the above lncRNA expression in the human pancreatic ductal cell line and three pancreatic cancer cell lines. The results showed that CASC8 was overexpressed in all pancreatic cancer lines, which indicated that CACS8 may also play a key role in promoting pancreatic cancer progression.

Recent research showed that m5C-related genes could regulate tumor immune microenvironment in breast cancer (40). LncRNAs are also known to be involved in regulating tumor-infiltrating lymph cells in malignant tumors (41).

In our study, we carefully evaluated the relationship between m5C-related lncRNAs and PDAC-infiltrating immune cells. Our results exhibited that the distribution of tumor-infiltrating immune cells in PDAC were different in high- or low-risk groups. The immune scores and stromal scores of the high-risk group were also lower than the low-risk group. We found that naïve B cells, CD8+T cells, Treg cells, and resting NK cells had a higher expression level in the low-risk group. CD8+T cells, Treg cells, and NK cells were considered as tumor suppressors in malignant tumors. In pancreatic cancer, the depletion of Treg cells made the abnormal distribution of fibroblast, which could recruit myeloid cells and restore the immune suppression environment *via* secreting multiple cell chemokine including CCL3, CCL6, and CCL8 (42). In addition, the M0 and M2 phenotype macrophages had a higher expression level in the high-risk group. Based on the previous study, M2 phenotype macrophage has been proven as a co-carcinogenic factor in pancreatic cancer (43). Therefore, m5C-related lncRNAs may regulate pancreatic cancer progression *via* promoting M2 phenotype macrophage polarization or infiltration in PDAC. As we know, there were no published reports about detecting the potential function of m5C-related lncRNAs in regulating the distribution of tumor-infiltrating lymph cells in PDAC at present.

However, there are still some limitations in our study. For example, all the PDAC patients’ transcriptome expression data and clinical information were downloaded and analyzed from the TCGA database or GEPIA online database. We only evaluated the m5C-related lncRNA risk model *via* internal validation, lacking external database validation. Nevertheless, we tried to download the PDAC patients’ information from ICGC (International Cancer Genome Consortium) or GEO database, but the corresponding risk scores could not be calculated due to the lack of expression data of the selected m5C-related lncRNAs in these databases. In addition, we only detected the screened m5C-related lncRNA expression *in vitro*, and the *in vivo* experiments and PDAC patients’ tumor tissue validation should still be performed in the future to make the prediction results more reliable. Moreover, the nomogram we constructed only calibrated within 3 years, because of the small number of PDAC patients and the lack of T1 stage patients, which suggested that our findings need further validation in larger PDAC patient follow-up cohorts. Additionally, there was a lack of molecular mechanism research in our study. Identifying which m5C-related lncRNAs could regulate pancreatic cancer survival is just the beginning. We will explore the specific mechanism of the screened lncRNAs that affected PDAC progression in our next work.

## Conclusion

We constructed an 8-m5C-related lncRNA prognostic risk model for PDAC patients based on the transcriptome expression and clinical data from TCGA. The m5C-related lncRNA risk model was proved to have an independent prognostic value and provided an accurate survival prediction for PDAC patients. In addition, our research also offered us a better understanding of the regulation of tumor-infiltrating lymph cells in PDAC. In brief, the m5C-related lncRNA risk model may provide us the potential biomarkers or treatment targets for PDAC.

## Data Availability Statement

The datasets presented in this study can be found in online repositories. The names of the repository/repositories and accession number(s) can be found in the article/**Supplementary Material**.

## Ethics Statement

All the patient’s raw data in our study were downloaded from TCGA, so there are no ethical issues and other conflicts of interest. The patients/participants provided their written informed consent to participate in this study.

## Author Contributions

HY, JL, and LZ summarized and analyzed the TCGA patients’ data. HY and PW drafted the manuscript. KJ and BX designed and monitored the research. All authors contributed to the article and approved the submitted version.

## Funding

This research was funded by the National Natural Science Foundation of China (Nos. 82072706 and 81871980) and Jiangsu Key Medical Discipline (General Surgery, ZDXKA2016005).

## Conflict of Interest

The authors declare that the research was conducted in the absence of any commercial or financial relationships that could be construed as a potential conflict of interest.

## Publisher’s Note

All claims expressed in this article are solely those of the authors and do not necessarily represent those of their affiliated organizations, or those of the publisher, the editors and the reviewers. Any product that may be evaluated in this article, or claim that may be made by its manufacturer, is not guaranteed or endorsed by the publisher.

## Supplementary Material

Supplementary Figure 1 Detecting the expression of m5C regulators *via* GEPIA online database. *P < 0.05.

Supplementary Figure 2 The overall survival in other clinical factors subgroups.

Supplementary Figure 3 Heatmaps showed the differential expression of the 8 m5C-related lncRNAs in subgroups. Scatter plot revealed the risk score location of high-risk and low-risk PDAC patients and the relationship between survival time and risk score in subgroups (**A**: Group **A**; **B**: Group **B**).

## References

[1] Vincent A Herman J Schulick R Hruban RH Goggins M. Pancreatic Cancer. CA Cancer J Clin (2011) 378:607–20. doi: 10.1016/S0140-6736(10)62307-0 PMC3062508 21620466

[2] Siegel RL Miller KD Fuchs HE Jemal A. Cancer Statistics, 2021. CA Cancer J Clin (2021) 71:7–33. doi: 10.3322/caac.21654 33433946

[3] Schizas D Charalampakis N Kole C Economopoulou P Koustas E Gkotsis E. Immunotherapy for Pancreatic Cancer: A 2020 Update. Cancer Treat Rev (2020) 86:102016. doi: 10.1016/j.ctrv.2020.102016 32247999

[4] Jain T Dudeja V. The War Against Pancreatic Cancer in 2020 - Advances on All Fronts. Nat Rev Gastroenterol Hepatol (2021) 18:99–100. doi: 10.1038/s41575-020-00410-4 33414515

[5] Kaliman P. Epigenetics and Meditation. Curr Opin Psychol (2019) 28:76–80. doi: 10.1016/j.copsyc.2018.11.010 30522005

[6] Kanwal R Gupta K Gupta S. Cancer Epigenetics: An Introduction. Methods Mol Biol (Clifton NJ) (2015) 1238:3–25. doi: 10.1007/978-1-4939-1804-1_1 25421652

[7] Ma S Chen C Ji X Liu J Zhou Q Wang G. The Interplay Between M6a RNA Methylation and Noncoding RNA in Cancer. J Hematol Oncol (2019) 12:121. doi: 10.1186/s13045-019-0805-7 31757221 PMC6874823

[8] He L Li H Wu A Peng Y Shu G Yin G. Functions of N6-Methyladenosine and its Role in Cancer. Mol Cancer (2019) 18:176. doi: 10.1186/s12943-019-1109-9 31801551 PMC6892141

[9] Chellamuthu A Gray SG. The RNA Methyltransferase NSUN2 and Its Potential Roles in Cancer. Cells (2020) 9. doi: 10.3390/cells9081758 PMC7463552 32708015

[10] Nombela P Miguel-López B Blanco S. The Role of M(6)A, M(5)C and Ψ RNA Modifications in Cancer: Novel Therapeutic Opportunities. Mol Cancer (2021) 20:18. doi: 10.1186/s12943-020-01263-w 33461542 PMC7812662

[11] Chen X Li A Sun BF Yang Y Han YN Yuan X. 5-Methylcytosine Promotes Pathogenesis of Bladder Cancer Through Stabilizing mRNAs. Nat Cell Biol (2019) 21:978–90. doi: 10.1038/s41556-019-0361-y 31358969

[12] Yang R Liang X Wang H Guo M Shen H Shi Y. The RNA Methyltransferase NSUN6 Suppresses Pancreatic Cancer Development by Regulating Cell Proliferation. EBioMedicine (2021) 63:103195. doi: 10.1016/j.ebiom.2020.103195 33418496 PMC7804980

[13] Yu X Zhang Q Gao F Zhang M Zheng Q He Y. Predictive Value of M5c Regulatory Gene Expression in Pancreatic Adenocarcinoma. Sci Rep (2021) 11:17529. doi: 10.1038/s41598-021-96470-w 34471186 PMC8410865

[14] Pan J Huang Z Xu Y. M5c-Related lncRNAs Predict Overall Survival of Patients and Regulate the Tumor Immune Microenvironment in Lung Adenocarcinoma. Front Cell Dev Biol (2021) 9:671821. doi: 10.3389/fcell.2021.671821 34268304 PMC8277384

[15] Qian X Zhao J Yeung PY Zhang QC Kwok CK. Revealing lncRNA Structures and Interactions by Sequencing-Based Approaches. Trends Biochem Sci (2019) 44:33–52. doi: 10.1016/j.tibs.2018.09.012 30459069

[16] Bridges MC Daulagala AC Kourtidis A. LNCcation: lncRNA Localization and Function. J Cell Biol (2021) 220. doi: 10.1083/jcb.202009045 PMC7816648 33464299

[17] Sun T Wu Z Wang X Wang Y Hu X Qin W. LNC942 Promoting METTL14-Mediated M(6)A Methylation in Breast Cancer Cell Proliferation and Progression. Oncogene (2020) 39:5358–72. doi: 10.1038/s41388-020-1338-9 32576970

[18] Arneth B. Tumor Microenvironment. Medicina (Kaunas Lithuania) (2019) 56(1):15. doi: 10.3390/medicina56010015 PMC7023392 31906017

[19] Hinshaw DC Shevde LA. The Tumor Microenvironment Innately Modulates Cancer Progression. Cancer Res (2019) 79:4557–66. doi: 10.1158/0008-5472.CAN-18-3962 PMC6744958 31350295

[20] Huang JK Ma L Song WH Lu BY Huang YB Dong HM. LncRNA-MALAT1 Promotes Angiogenesis of Thyroid Cancer by Modulating Tumor-Associated Macrophage FGF2 Protein Secretion. J Cell Biochem (2017) 118:4821–30. doi: 10.1002/jcb.26153 28543663

[21] Xu M Xu X Pan B Chen X Lin K Zeng K. LncRNA SATB2-AS1 Inhibits Tumor Metastasis and Affects the Tumor Immune Cell Microenvironment in Colorectal Cancer by Regulating SATB2. Mol Cancer (2019) 18:135. doi: 10.1186/s12943-019-1063-6 31492160 PMC6729021

[22] Wei C Liang Q Li X Li H Liu Y Huang X. Bioinformatics Profiling Utilized a Nine Immune-Related Long Noncoding RNA Signature as a Prognostic Target for Pancreatic Cancer. J Cell Biochem (2019) 120:14916–27. doi: 10.1002/jcb.28754 31016791

[23] Lin QJ Yang F Jin C Fu DL. Current Status and Progress of Pancreatic Cancer in China. World J Gastroenterol (2015) 21:7988–8003. doi: 10.3748/wjg.v21.i26.7988 26185370 PMC4499341

[24] Mongan NP Emes RD Archer N. Detection and Analysis of RNA Methylation. F1000Research (2019) 26:8. doi: 10.12688/f1000research.17956.1 PMC6489984 31069058

[25] Traube FR Carell T. The Chemistries and Consequences of DNA and RNA Methylation and Demethylation. RNA Biol (2017) 14:1099–107. doi: 10.1080/15476286.2017.1318241 PMC5699545 28440690

[26] Xie S Chen W Chen K Chang Y Yang F Lin A. Emerging Roles of RNA Methylation in Gastrointestinal Cancers. Cancer Cell Int (2020) 20:585. doi: 10.1186/s12935-020-01679-w 33372610 PMC7720447

[27] Li Q Li X Tang H Jiang B Dou Y Gorospe M. NSUN2-Mediated M5c Methylation and METTL3/METTL14-Mediated M6a Methylation Cooperatively Enhance P21 Translation. J Cell Biochem (2017) 118:2587–98. doi: 10.1002/jcb.25957 PMC5509477 28247949

[28] Bohnsack KE Höbartner C Bohnsack MT. Eukaryotic 5-Methylcytosine (M^5^C) RNA Methyltransferases: Mechanisms, Cellular Functions, and Links to Disease. Genes (2019) 10(2):102. doi: 10.3390/genes10020102 PMC6409601 30704115

[29] Zhao BS Roundtree IA He C. Post-Transcriptional Gene Regulation by mRNA Modifications. Nat Rev Mol Cell Biol (2017) 18:31–42. doi: 10.1038/nrm.2016.132 27808276 PMC5167638

[30] Nandwani A Rathore S Datta M. LncRNAs in Cancer: Regulatory and Therapeutic Implications. Cancer Lett (2021) 501:162–71. doi: 10.1016/j.canlet.2020.11.048 33359709

[31] Gil N Ulitsky I. Regulation of Gene Expression by Cis-Acting Long Non-Coding RNAs. Nat Rev Genet (2020) 21:102–17. doi: 10.1038/s41576-019-0184-5 31729473

[32] Kopp F Mendell JT. Functional Classification and Experimental Dissection of Long Noncoding RNAs. Cell (2018) 172:393–407. doi: 10.1016/j.cell.2018.01.011 29373828 PMC5978744

[33] Best MG Wesseling P Wurdinger T. Tumor-Educated Platelets as a Noninvasive Biomarker Source for Cancer Detection and Progression Monitoring. Cancer Res (2018) 78:3407–12. doi: 10.1158/0008-5472.CAN-18-0887 29921699

[34] Soria F Krabbe LM Todenhöfer T Dobruch J Mitra AP Inman BA. Molecular Markers in Bladder Cancer. World J Urol (2019) 37:31–40. doi: 10.1007/s00345-018-2503-4 30259123 PMC6510866

[35] Chandra Gupta S Nandan Tripathi Y. Potential of Long Non-Coding RNAs in Cancer Patients: From Biomarkers to Therapeutic Targets. Int J Cancer (2017) 140:1955–67. doi: 10.1002/ijc.30546 27925173

[36] Li Y Jiang T Zhou W Li J Li X Wang Q. Pan-Cancer Characterization of Immune-Related lncRNAs Identifies Potential Oncogenic Biomarkers. Nat Commun (2020) 11:1000. doi: 10.1038/s41467-020-14802-2 32081859 PMC7035327

[37] Jiang X Guan J Xu Y Ren H Jiang J Wudu M. Silencing of CASC8 Inhibits Non-Small Cell Lung Cancer Cells Function and Promotes Sensitivity to Osimertinib *via* FOXM1. J Cancer (2021) 12:387–96. doi: 10.7150/jca.47863 PMC7739000 33391435

[38] Haerian MS Haerian BS Molanaei S Kosari F Sabeti S Bidari-Zerehpoosh F. Lack of Association of CASC8 Rs1447295 With Colorectal Cancer in Iranian Population: A Multicenter Case-Control Study. Gene (2017) 634:74–6. doi: 10.1016/j.gene.2017.08.042 28887158

[39] Wu ER Hsieh MJ Chiang WL Hsueh KC Yang SF Su SC. Association of lncRNA CCAT2 and CASC8 Gene Polymorphisms With Hepatocellular Carcinoma. Int J Environ Res Public Health (2019) 16(16):2833. doi: 10.3390/ijerph16162833 PMC6720737 31398859

[40] Huang Z Pan J Wang H Du X Xu Y Wang Z. Prognostic Significance and Tumor Immune Microenvironment Heterogenicity of M5c RNA Methylation Regulators in Triple-Negative Breast Cancer. Front Cell Dev Biol (2021) 9:657547. doi: 10.3389/fcell.2021.657547 33928086 PMC8076743

[41] Sun J Zhang Z Bao S Yan C Hou P Wu N. Identification of Tumor Immune Infiltration-Associated lncRNAs for Improving Prognosis and Immunotherapy Response of Patients With Non-Small Cell Lung Cancer. J Immunother Cancer (2020) 8(1):e000110. doi: 10.1136/jitc-2019-000110 32041817 PMC7057423

[42] Zhang Y Lazarus J Steele NG Yan W Lee HJ Nwosu ZC. Regulatory T-Cell Depletion Alters the Tumor Microenvironment and Accelerates Pancreatic Carcinogenesis. Cancer Discov (2020) 10:422–39. doi: 10.1158/2159-8290.CD-19-0958 PMC7224338 31911451

[43] Yang J Li Y Sun Z Zhan H. Macrophages in Pancreatic Cancer: An Immunometabolic Perspective. Cancer Lett (2021) 498:188–200. doi: 10.1016/j.canlet.2020.10.029 33122097

