# Supplementary figures and images for: Prognostic Risk Model and Tumor Immune Environment Modulation of m5C-Related LncRNAs in Pancreatic Ductal Adenocarcinoma
Source: Front Immunol. 2021 Dec 8;12:800268. doi: 10.3389/fimmu.2021.800268 (PMC8692582; doi:10.3389/fimmu.2021.800268)

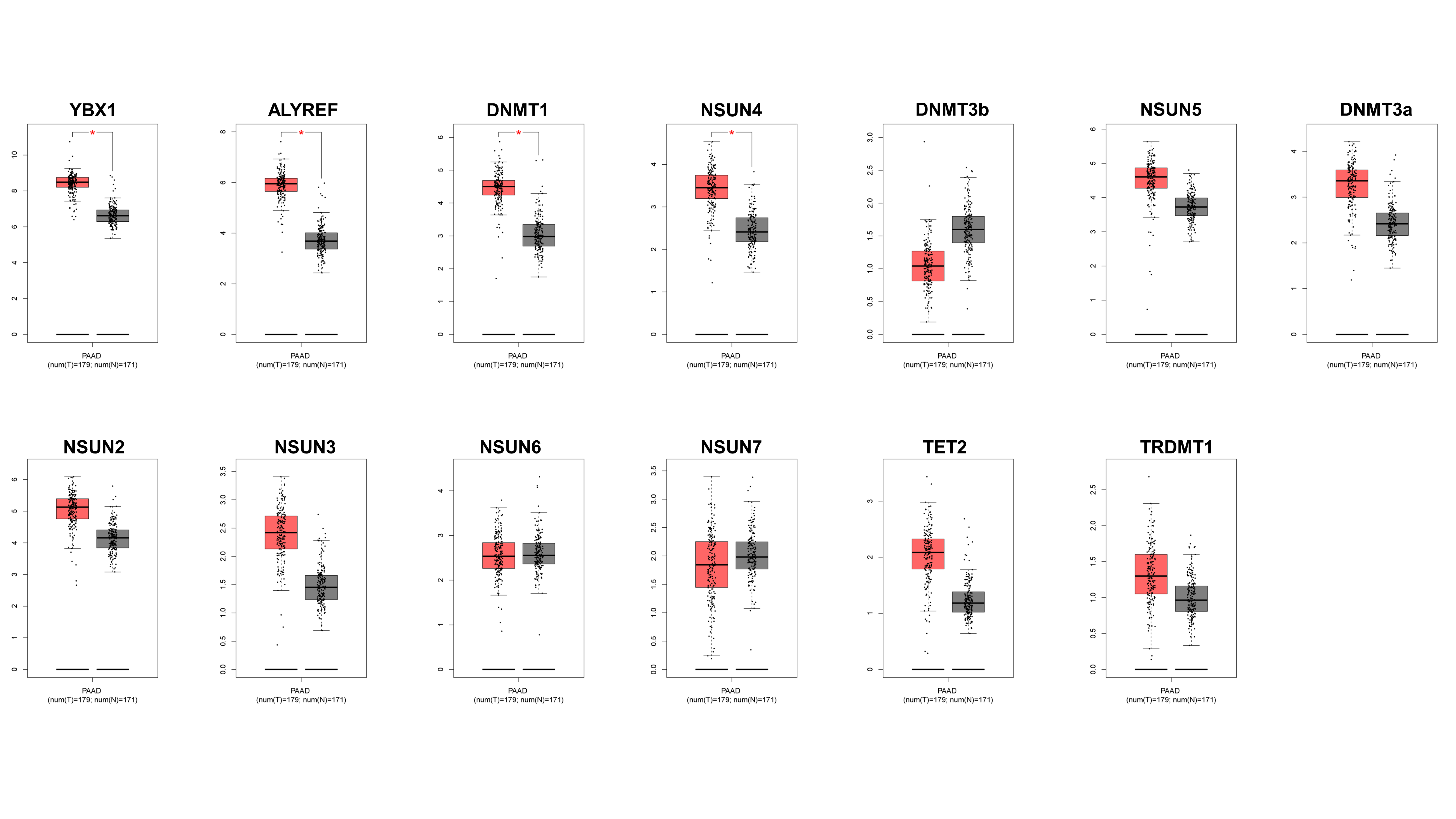

Supplement: Supplementary Figure 1 — Detecting the expression of m5C regulators via GEPIA online database. *P < 0.05. [file Image_1.tif]

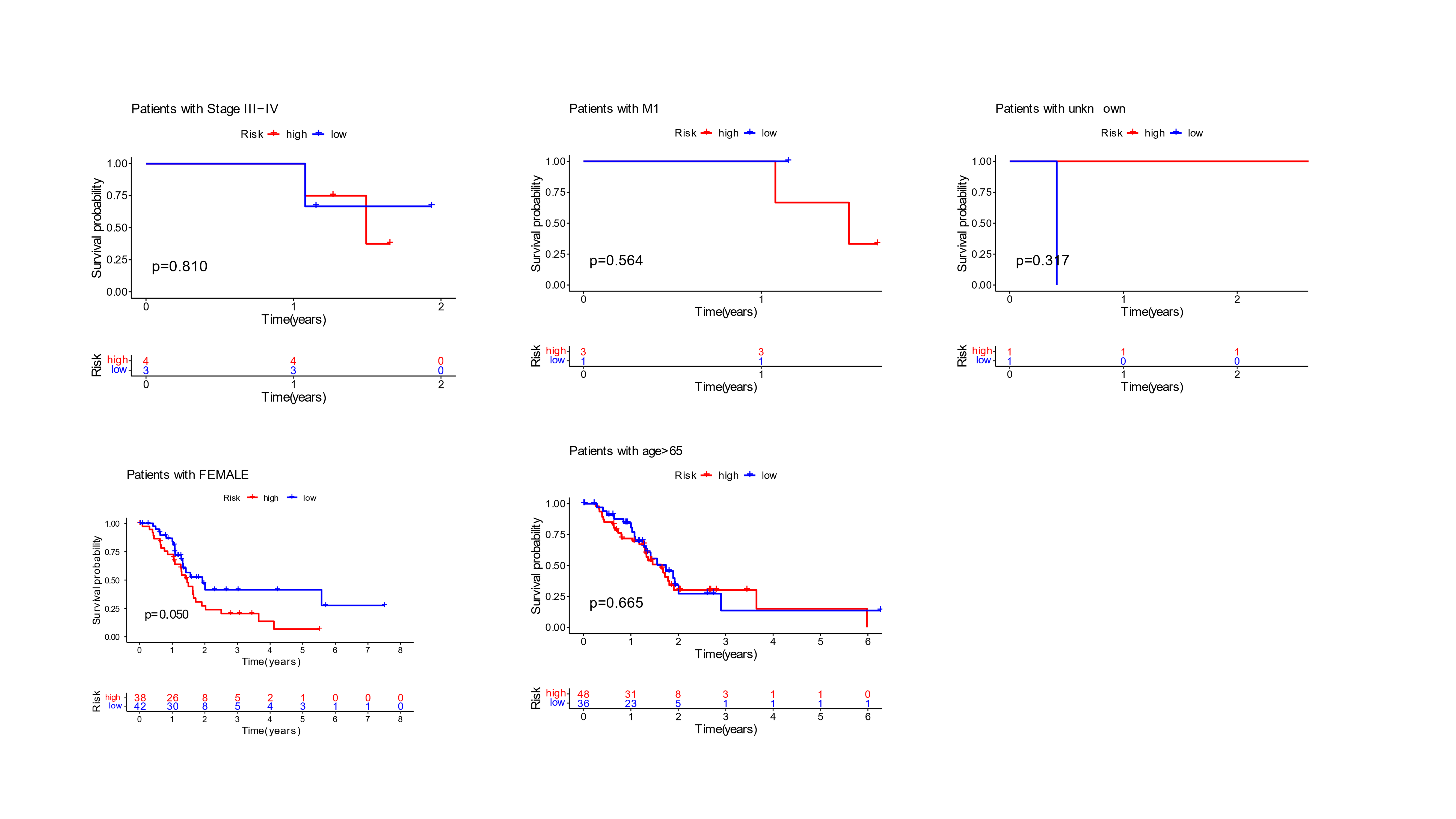

Supplement: Supplementary Figure 2 — The overall survival in other clinical factors subgroups. [file Image_2.tif]

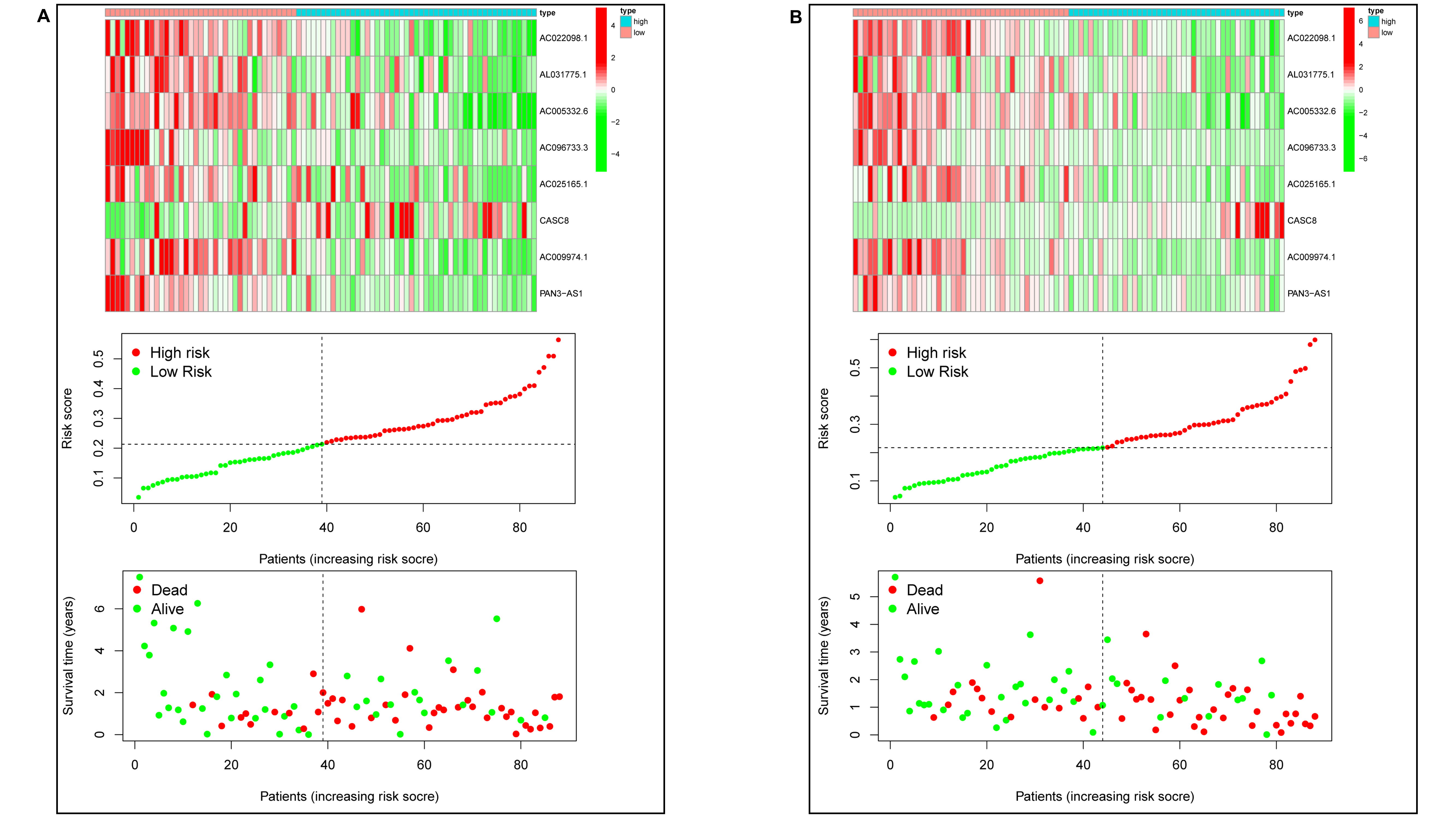

Supplement: Supplementary Figure 3 — Heatmaps showed the differential expression of the 8 m5C-related lncRNAs in subgroups. Scatter plot revealed the risk score location of high-risk and low-risk PDAC patients and the relationship between survival time and risk score in subgroups (A: Group A; B: Group B). [file Image_3.tif]

**Table.2 Flowchart of the data collection and analysis**

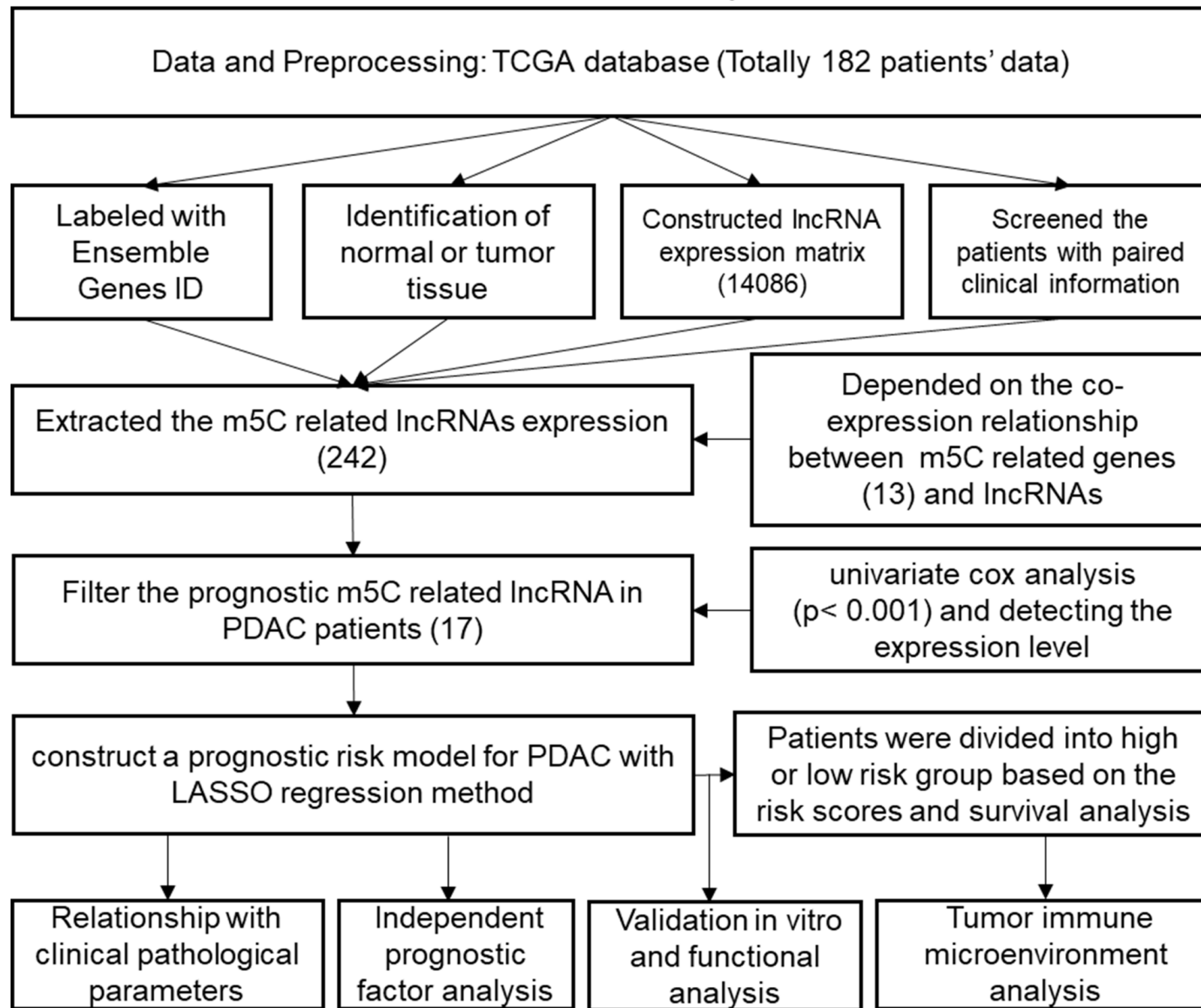

Supplement: Supplementary file 5 [file Table_2.pdf]
